# Impacto de la ansiedad dental en la calidad de vida relacionada con la salud bucal de adultos mayores en zona rural del Perú

**DOI:** 10.21142/2523-2754-1301-2025-229

**Published:** 2025-03-03

**Authors:** Soledad Jimena Juárez Pacheco, María Alejandra Juárez Pacheco, Gabriela Mariana Castro-Núñez, Enrique Manuel de los Ríos Fernández, Mauro Henrique Nogueira Guimarães de Abreu, Wilfredo Gustavo Escalante-Otárola

**Affiliations:** 1 Facultad de Odontología, Universidad Católica de Santa María. Arequipa, Perú. jimenajuarez543@gmail.com maria.juarezp@ucsm.edu.pe gcastron@ucsm.edu.pe edelosrios@ucsm.edu.pe wilfredo.escalante@ucsm.edu.pe Universidad Católica de Santa María Facultad de Odontología Universidad Católica de Santa María Arequipa Peru jimenajuarez543@gmail.com maria.juarezp@ucsm.edu.pe gcastron@ucsm.edu.pe edelosrios@ucsm.edu.pe wilfredo.escalante@ucsm.edu.pe; 2 Faculdade de Odontologia, Universidade Federal de Minas Gerais. Belo Horizonte, Brasil. maurohenriqueabreu@gmail.com Universidade Federal de Minas Gerais Faculdade de Odontologia Universidade Federal de Minas Gerais Belo Horizonte Brazil maurohenriqueabreu@gmail.com

**Keywords:** ansiedad dental, calidad de vida, salud bucal, adultos mayores, barreras de acceso, dental anxiety, quality of life, oral health, older adults, access barriers

## Abstract

**Objetivo:**

Determinar la prevalencia de problemas bucales y ansiedad dental en adultos mayores rurales, y su impacto en la calidad de vida relacionada con la salud bucal (OHRQoL).

**Metodología:**

Se realizó un estudio descriptivo y analítico transversal en adultos mayores de San Juan de Siguas, Arequipa, con 64 participantes mayores de 60 años que dieron su consentimiento informado. Se recolectaron datos sociodemográficos, clínicos y de salud bucal mediante entrevistas estructuradas, utilizando la Escala de Ansiedad Dental Modificada (MDAS) y el cuestionario OHIP-5 para medir ansiedad dental y calidad de vida bucal (OHRQoL), respectivamente. Se aplicó regresión logística binaria para analizar la relación entre ansiedad dental y calidad de vida bucal, ajustando por variables relevantes.

**Resultados:**

Los participantes tenían una edad promedio de 68,5 años, la mayoría eran mujeres (54,7%) y carecían de seguro de salud (85,9%). Todos tenían dientes cariados y el 68,8% había perdido piezas dentales. El 50% presentó ansiedad dental moderada (promedio MDAS: 11,6), y el impacto en la calidad de vida bucal fue moderado (promedio OHIP-5: 8,4). El modelo logístico indicó que el aumento de una unidad en la ansiedad dental (MDAS) incrementaba 1,49 veces la chance de un mayor impacto en la calidad de vida bucal. Además, los participantes satisfechos con la atención odontológica tenían 4,67 veces más chance de reportar un mayor impacto.

**Conclusiones:**

La ansiedad dental empeora la OHRQoL en adultos mayores rurales, debido a barreras de acceso y experiencias negativas en la atención.

## Abstract (en)

**Objective:**

To determine the prevalence of oral health problems and dental anxiety in rural older adults, and their impact on oral health-related quality of life (OHRQoL).

**Methods:**

A cross-sectional descriptive and analytical study was conducted among older adults in San Juan de Siguas, Arequipa, involving 64 participants aged 60 and above who provided informed consent. Sociodemographic, clinical, and oral health data were collected through structured interviews, using the Modified Dental Anxiety Scale (MDAS) and the OHIP-5 questionnaire to assess dental anxiety and OHRQoL, respectively. Binary logistic regression was applied to analyze the relationship between dental anxiety and OHRQoL, adjusting for relevant variables.

**Results:**

Participants had an average age of 68.5 years, the majority were women (54.7%), and most lacked health insurance (85.9%). All participants had decayed teeth, and 68.8% had experienced tooth loss. Half of the participants exhibited moderate dental anxiety (mean MDAS: 11.6), and the impact on OHRQoL was moderate (mean OHIP-5: 8.4). Logistic regression revealed that each one-unit increase in dental anxiety (MDAS) increased the odds of a greater impact on OHRQoL by 1.49 times. Additionally, participants satisfied with dental care were 4.67 times more likely to report a greater impact**.**

**Conclusions:**

Dental anxiety worsens OHRQoL in rural older adults due to access barriers and negative dental care experiences.

INTRODUCCIÓN

La salud bucal es un componente esencial para un envejecimiento saludable, ya que influye de manera significativa en la salud sistémica, cognitiva y psicológica de los adultos mayores ^1^. Una deficiente salud bucal puede repercutir negativamente en su calidad de vida, lo que subraya la importancia de enfoques de atención integrados y multidisciplinarios para esta población ^2^. En este contexto, vivir en zonas rurales representa un factor de riesgo adicional, ya que los residentes rurales suelen presentar tasas más elevadas de pérdida dental y menor acceso a servicios odontológicos adecuados ^3^. 

A medida que los adultos mayores envejecen, experimentan un deterioro más acelerado de su salud bucal, en parte debido a factores sistémicos como la hiposalivación, que agrava los problemas bucales existentes ^4^. Además, la ansiedad dental -que se define como una actitud negativa frente al tratamiento odontológico- desempeña un papel importante en el comportamiento de los pacientes, que afecta la frecuencia del cepillado dental y las visitas al odontólogo ^5^^,^^6^. A pesar de que la ansiedad dental es común, a menudo pasa desapercibida en los adultos mayores, lo que dificulta su manejo oportuno ^7^.

En el Perú, a pesar de la implementación del aseguramiento universal en salud, el acceso y uso de servicios de salud bucal entre los adultos mayores sigue siendo limitado, con disparidades marcadas por factores socioeconómicos y geográficos ^8^^,^^9^. La ansiedad dental en esta población tiende a estar asociada con una mayor preocupación por el envejecimiento, pero puede ser atenuada mediante intervenciones de rehabilitación oral que mejoren la calidad de vida relacionada con la salud bucal (OHRQoL) ^10^. 

Aunque la ansiedad dental en adultos mayores de zonas rurales representa desafíos significativos, también abre la puerta a oportunidades para desarrollar intervenciones innovadoras y adaptadas a las necesidades de esta población. Comprender cómo la ansiedad dental afecta la salud bucal es esencial para diseñar estrategias que aborden tanto los aspectos clínicos como los emocionales de su cuidado. Este estudio busca determinar la prevalencia de problemas de salud bucal y ansiedad dental en adultos mayores rurales, así como su impacto en la calidad de vida relacionada con la salud bucal (OHRQoL). Los hallazgos proporcionarán evidencia crítica para identificar barreras específicas en el acceso a servicios odontológicos, y servirán como base para el desarrollo de programas de intervención personalizados que mejoren la salud bucal y reduzcan la ansiedad dental en comunidades rurales. Asimismo, estos resultados contribuirán a la formulación de políticas públicas destinadas a cerrar las brechas en el acceso a la atención odontológica, para promover un envejecimiento saludable y el bienestar integral de esta población vulnerable.

## MATERIAL Y MÉTODOS

Este estudio descriptivo transversal, aprobado por el Comité de Ética de la Universidad Católica de Santa María (código: 107-2022), evaluó la asociación entre la ansiedad dental y la calidad de vida relacionada con la salud bucal en adultos mayores del distrito de San Juan de Siguas (Arequipa), una zona rural del Perú. Los participantes, seleccionados de una población censada de 80 adultos mayores, según el INEI (2017) ^11^, incluyeron a 64 personas mayores de 60 años, capaces de comunicarse y dispuestas a participar, lo que garantiza un intervalo de confianza del 95% y un margen de error del 5%. 

Previo al inicio, los participantes otorgaron su consentimiento informado, en el que se explicaron los objetivos del estudio, los detalles de la participación, los riesgos y beneficios, y se aseguró además la confidencialidad y el anonimato de los datos. La recolección de información se realizó mediante entrevistas estructuradas que abarcaron tres áreas principales: datos sociodemográficos, como edad, sexo, estado civil, nivel educativo e ingreso económico; datos clínicos, incluyendo diagnósticos médicos, problemas de salud crónicos, problemas bucales e historial de tabaquismo; y prácticas de salud bucal, como hábitos de higiene oral, visitas al dentista y percepción de la salud bucal. 

Para medir las variables principales, se utilizaron dos instrumentos estandarizados. La Escala de Ansiedad Dental Modificada (MDAS) ^12^ evaluó los niveles de ansiedad mediante cinco preguntas sobre reacciones frente a situaciones odontológicas, con una puntuación total entre 5 y 25, clasificada en cinco niveles de ansiedad. El cuestionario OHIP-5 ^13^, compuesto por cinco preguntas, midió la OHRQoL en el último mes, también con puntuaciones de 5 a 25, donde valores más altos indican un mayor impacto negativo. 

Antes de la recolección oficial de datos, se llevó a cabo una prueba piloto con 10 adultos mayores, excluidos de la muestra principal, para evaluar la claridad, validez y consistencia del estudio. Estos participantes completaron anónimamente la entrevista estructurada, evaluando cada pregunta en una escala Likert del 1 al 5 y proporcionando comentarios que permitieron ajustes en las preguntas que obtuvieron puntuaciones de 3 o menos. Posteriormente, la recolección de datos, realizada en septiembre de 2023, fue llevada a cabo por una única investigadora, quien asistió a los participantes y verificó los datos sobre problemas de salud bucal. 

### Análisis estadístico

El análisis estadístico incluyó medidas descriptivas de tendencia central y variabilidad para las variables categóricas y cuantitativas. Se utilizaron modelos de regresión logística binaria para estimar odds ratios (OR) con intervalos de confianza al 95% y valores p, considerando el OHRQoL dicotomizado por la mediana como resultado y el MDAS como exposición principal. Las covariables, como edad, sexo, salario, nivel educativo, seguro de salud, satisfacción por atenciones previas y edentulismo, se incluyeron en el modelo ajustado si presentaron un valor p < 0,25, y se mantuvieron en el modelo final solo aquellas con p < 0,05. La calidad del modelo fue evaluada mediante la prueba de Hosmer-Lemeshow, mientras que la multicolinealidad se analizó con el factor de inflación de la varianza (VIF) y la distancia de Cook se utilizó para identificar posibles valores atípicos en los residuos.

## RESULTADOS

En este estudio participaron 64 adultos mayores del distrito de San Juan de Siguas, con una edad promedio de 68.5 ± 9.2 años. Según los datos demográficos presentados en la Tabla 1, el 54.7% de los participantes eran mujeres, el 59.4% tenía educación secundaria y el 48.4% era soltero. Además, solo el 14.1% de los participantes contaba con seguro de salud.

**Tabla 1:** Datos sociodemográficos de los adultos mayores

Datos sociodemográficos	N.º (64)	%
Sexo		
Masculino	29	45,3
Femenino	35	54,7
Edad		
60-69 años	44	68,8
70-79 años	10	15,6
80-89 años	7	10,9
90-99 años	3	4,7
Promedio ± DE	68,5 ± 9,2	
Nivel educativo		
Analfabeto(a)	5	7,8
Primaria	19	29,7
Secundaria	38	59,4
Técnico	1	1,6
Universitario	1	1,6
Estado civil		
Soltero(a)	31	48,4
Casado(a)	18	28,1
Divorciado(a)	3	4,7
Viudo(a)	12	18,8
Ingreso económico		
Suficiente	31	48,4
Insuficiente	33	51,6
Gastos de atención dental		
Sin seguro	55	85,9
Seguro de salud	9	14,1

DE Desviación estándar

La Tabla 2 muestra que los problemas crónicos más frecuentes fueron los desórdenes musculoesqueléticos (60.9%), la hipertensión arterial (43.8%) y los problemas oculares (43.8%). Además, el 76.6% de los participantes reportó haber contraído COVID-19.

**Tabla 2:** Datos clínicos de los adultos mayores

Datos clínicos	N.º (64)	%
Problemas crónicos de salud #		
No	5	7,8
Hipertensión arterial	28	43,8
Diabetes mellitus	9	14,1
Enfermedades respiratorias	9	14,1
Enfermedades cardiovasculares	14	21,9
Desórdenes gastrointestinales	20	31,3
Desórdenes renales	2	3,1
Desórdenes musculoesqueléticos	39	60,9
Problemas oculares	28	43,8
Trastornos mentales	2	3,1
COVID-19	49	76,6
Problemas de salud bucal #		
Dientes perdidos	44	68,8
Dientes cariados	64	100,0
Cálculo dental	63	98,4
Placa dental	62	96,9
Alteraciones en labios y/o lengua	4	6,3
Tabaco		
No fumador	60	93,8
Fumador	2	3,1
Exfumador	2	3,1

# Respuestas múltiples

En cuanto a la salud bucal, según los datos de la Tabla 3, todos los participantes presentaban dientes cariados, el 96.9% tenía cálculo o placa dental, y el 68.8% había perdido dientes. No obstante, el 93.8% de los participantes declaró no ser fumador.

**Tabla 3:** Prácticas de cuidado de su salud bucal y satisfacción con citas dentales de los adultos mayores

Variables	N.º (64)	%
Cepillado dental		
Sí	53	82,8
No	11	17,2
Frecuencia diaria del cepillado dental	N.º 53	
Una vez	9	17,0
Dos veces	42	79,2
Tres veces o más	2	3,8
Tiempo desde la última visita al dentista		
Últimos 6 meses	7	10,9
Entre 6 y 12 meses	22	34,4
Entre 12 y 24 meses	11	17,2
Más de 24 meses	24	37,5
Evaluación de visitas previas al dentista		
Satisfecho	32	50,0
Insatisfecho	32	50,0
Razones de la insatisfacción en visitas previas al dentista #	N.º 32	
Largo tiempo de espera	20	62,5
Inconvenientes con las citas	32	100,0
Mala calidad en la atención brindada	31	96,9
Desatención de quejas de los adultos mayores	1	3,1
Insuficiente información proporcionada	19	59,4

# Respuestas múltiples

En relación con la higiene oral, la Tabla 3 indica que el 82.8% de los adultos mayores se cepillaban los dientes, y la mayoría de ellos (79.2%) lo hacía dos veces al día. Sin embargo, el 37.5% no había visitado al dentista en los últimos 24 meses, y la mitad de los participantes (50%) expresó insatisfacción con sus citas odontológicas previas. Las razones de insatisfacción más comunes fueron los inconvenientes con las citas (100%), la mala calidad en la atención (96.9%), los tiempos de espera largos (62.5%) y la información insuficiente (59.4%). 

Por su parte, la Tabla 4 muestra que el 71.9% de los participantes percibe que su salud general es más importante que su salud bucal, mientras que el 79.7% considera que su salud bucal es regular. En cuanto al miedo y la ansiedad dental, las razones más comunes fueron malas experiencias previas (60.9%) y dolor durante los procedimientos (54.7%).

**Tabla 4:** Percepción y miedo asociados a la salud bucal de los adultos mayores

Variables	N.º (64)	%
Percepción del valor de la salud bucal		
La salud bucal es parte de la salud general.	16	25,0
La salud general es más importante que la salud bucal.	46	71,9
La salud bucal es más importante que la salud general.	2	3,1
Percepción del estado de su salud bucal		
Bueno	3	4,7
Regular	51	79,7
Malo	10	15,6
Motivos de miedo/ansiedad dental a citas dentales #		
Miedo a la infección	1	1,6
Miedo al sangrado	1	1,6
Vergüenza por los dientes	10	15,6
Dolor durante procedimientos dentales	35	54,7
Mala experiencia previa	39	60,9
Sin miedo/ansiedad dental	1	1,6

# Respuestas múltiples

Respecto de la ansiedad dental, la Tabla 5 revela que el 50% de los participantes experimentó ansiedad dental moderada, el 34.4% mostró baja ansiedad y el 12.5% presentó alta ansiedad, con un puntaje promedio en la MDAS de 11.6 ± 3.0.

**Tabla 5:** Puntaje en la escala de ansiedad dental modificada (MDAS) de los adultos mayores

Puntaje en escala de ansiedad dental	N.º (64)	%
Sin ansiedad	1	1,6
Baja ansiedad	22	34,4
Moderada ansiedad	32	50,0
Alta ansiedad	8	12,5
Extrema ansiedad (fobia)	1	1,6
Puntaje promedio general MDAS* 11,6 ± 3,0			

* Mínimo = 5 y máximo = 25; 5 = sin ansiedad, 6-10 = baja ansiedad, 11-14 = moderada ansiedad, 15-18 = alta ansiedad y 19-25 = extrema ansiedad.

En relación con el impacto en la calidad de vida, la Tabla 6 muestra que las quejas más frecuentes fueron las dificultades para realizar actividades cotidianas (23.4%), la incomodidad por la apariencia dental (18.8%) y el menor sabor de la comida (17.2%). El puntaje promedio del cuestionario OHIP-5 fue de 8,4 ± 2,2, lo que sugiere un impacto moderado en la OHRQoL. 

**Tabla 6:** Impacto de la salud bucal en la calidad de vida (OHIP-5) de los adultos mayores

Variables	N.º (64)	%
Dificultad para masticar alimentos en problemas de dientes, boca y/o prótesis			
Nunca	29	45,3
Casi nunca	31	48,3
Ocasionalmente	4	6,3
Con frecuencia	0	0,0
Casi siempre	0	0,0
Dolor intenso en boca		
Nunca	34	53,1
Casi nunca	25	39,1
Ocasionalmente	3	4,7
Con frecuencia	2	3,1
Casi siempre	0	0,0
Incomodidad por apariencia en dientes, boca y/o prótesis			
Nunca	27	42,4
Casi nunca	25	39,1
Ocasionalmente	12	18,8
Con frecuencia	0	0,0
Casi siempre	0	0,0
Menor sabor de la comida por problemas en dientes, boca y/o prótesis			
Nunca	29	45,3
Casi nunca	23	35,9
Ocasionalmente	11	17,2
Con frecuencia	1	1,6
Casi siempre	0	0,0
Dificultad para realizar actividades cotidianas por problemas en dientes, boca y/o prótesis			
Nunca	29	45,3
Casi nunca	20	31,3
Ocasionalmente	15	23,4
Con frecuencia	0	0,0
Casi siempre	0	0,0

Finalmente, la Tabla 7 describe las covariables MDAS y edad en la población de adultos mayores de San Juan de Siguas.

**Tabla 7:** Descripción de covariables MDAS (escala de ansiedad dental modificada) y edad en adultos mayores, San Juan de Siguas, 2023

Covariables	Mediana	Mínimo	Máximo
MDAS	8	5	13
Edad	66	60	96

En la Tabla 8, se presentan los factores asociados con el impacto en la calidad de vida en esta población. El modelo final mostró que, por cada incremento de una unidad en el puntaje del MDAS, la chance de experimentar un alto impacto en la calidad de vida relacionada con la salud bucal aumentaba 1,49 veces (IC 95%; 1.15-1.93). Además, los participantes que reportaron estar satisfechos con la atención odontológica tenían 4,67 veces más chances (IC 95%; 1,33-16,35) de experimentar un alto impacto en su calidad de vida. La prueba de Hosmer y Lemeshow confirmó la adecuación del modelo final (p = 0,372), mientras que la evaluación de residuos mostró valores adecuados, todos inferiores a 1, y los valores de VIF cercanos a la unidad descartaron problemas de multicolinealidad.

**Tabla 8:** Factores asociados al impacto en la calidad de vida (dicotomizada por la mediana), San Juan de Siguas, 2023

Covariables	OHRQOL superior a la mediana (%)	OR (IC 95%) no ajustada	Valor p	OR (IC 95%) ajustada	Valor p
MDAS		1,43 (1,13-1,79)	0,002	1,49 (1,15-1,93)	0,002
Edad		1,01 (0,95-1,07)	0,779		
Sexo					
Femenino	37,1	1,12 (0,40-3,14)	0,825		
Masculino	34,5	1			
Salario					
Suficiente	35,5	1	0,942		
Insuficiente	36,4	1,04 (0,37-2,89)			
Grado de Instrucción					
Sin/primaria	29,2	1	0,384		
Secundaria/superior	40,0	1,62 (0,55-4,79)			
Seguro de salud					
Tiene	33,3	1	0,861		
No tiene	36,4	1,14 (0,26-5,08)			
Satisfacción previa					
No	21,9	1	0,022	1	0,016
Sí	50,0	3,57 (1,20-10,60)		4,67 (1,33-16,35)	
Edentulismo					
No	25,0	1	0,224		
Sí	40,9	2,08 (0,64-6,74)			

## DISCUSIÓN

El presente estudio evidencia que los adultos mayores de San Juan de Siguas enfrentan una elevada carga de enfermedades crónicas, con una alta prevalencia de desórdenes musculoesqueléticos, hipertensión arterial y problemas oculares. En el ámbito de la salud bucal, se observan condiciones deficientes, sumadas a una insatisfacción notable con la atención odontológica recibida. A pesar de estas problemáticas, el impacto en la OHRQoL fue moderado. El modelo predictivo final indicó que la ansiedad dental y la satisfacción con la atención odontológica tienen un efecto significativo en esta dimensión de la calidad de vida.

Los adultos mayores en zonas rurales enfrentan una carga importante de enfermedades crónicas, agravada por barreras socioeconómicas y un acceso limitado a los servicios de salud ^14^. Nuestros resultados coinciden con investigaciones previas que señalan a la hipertensión como una de las condiciones crónicas más comunes entre adultos mayores de áreas rurales en Sudamérica ^15^. Además, afecciones musculoesqueléticas, como la artritis, y problemas visuales son muy prevalentes, lo que deteriora su estado de salud general y limita su funcionalidad diaria ^14^^,^^15^. Aunque las tasas de diagnóstico suelen ser más bajas en comparación con zonas urbanas, los adultos mayores en áreas rurales experimentan una progresión más rápida de estas enfermedades, lo que sugiere posibles casos de infradiagnóstico y un manejo clínico deficiente ^16^.

En cuanto a la salud bucal, los adultos mayores evaluados presentaron condiciones similares a las descritas en estudios internacionales, que documentan una mayor prevalencia de caries dental, enfermedades periodontales y edentulismo en zonas rurales frente a áreas urbanas ^17^^-^^19^. Factores como ingresos económicos, nivel educativo y ocupación desempeñan un rol crucial en estas disparidades de salud bucal ^17^^,^^18^^,^^20^. Además, las zonas rurales en Sudamérica enfrentan desafíos particulares, como una menor disponibilidad de centros de salud y profesionales capacitados, lo que se traduce en un menor uso de los servicios odontológicos ^21^. Este contexto explica, al menos parcialmente, la insatisfacción expresada por los participantes respecto de sus experiencias previas en citas odontológicas, probablemente vinculada con dificultades de acceso, percepciones de atención de baja calidad y resultados insatisfactorios. Nuestros resultados también muestran que los adultos mayores satisfechos con la atención odontológica tienen 4,67 veces más probabilidades de presentar un alto impacto en su calidad de vida, lo que refleja la compleja relación entre satisfacción y resultados percibidos en salud.

La salud bucal, esencial para el bienestar general, suele ser descuidada, especialmente en zonas rurales donde el acceso limitado a los servicios dentales y las dificultades socioeconómicas contribuyen a su deterioro. Problemas como xerostomía, dolor orofacial y dificultad para masticar han demostrado tener un impacto negativo en la calidad de vida relacionada con la salud bucal de los adultos mayores ^22^^-^^24^. En el Perú, estas limitaciones se ven agravadas por el aislamiento geográfico y la falta de infraestructura, lo que restringe aún más el acceso a la atención dental y a servicios preventivos en áreas rurales ^19^. Sin embargo, nuestros hallazgos destacan que, a pesar de las problemáticas descritas, el impacto en la OHRQoL fue moderado. Este resultado podría estar influido por factores individuales como resiliencia o una percepción menos crítica de su salud, aspectos que deben ser explorados con mayor profundidad en futuras investigaciones.

La ansiedad dental, un problema frecuente en esta población, emerge como un factor clave que afecta negativamente la calidad de vida relacionada con la salud bucal, dado que a menudo conduce a evitar la atención odontológica. Esta evitación agrava el deterioro de la salud bucal, lo que contribuye a condiciones desfavorables que repercuten en el bienestar general ^10^. Nuestro modelo predictivo mostró que un aumento en la ansiedad dental incrementa 1,49 veces la probabilidad de experimentar un alto impacto en la calidad de vida. Además, el acceso limitado a los servicios dentales, la infraestructura insuficiente y los malos hábitos de higiene oral intensifican los niveles de ansiedad dental y complican el manejo de la salud bucal en zonas rurales ^25^. 

Esta relación entre la ansiedad dental y la calidad de vida también está influenciada por factores estructurales, como el nivel socioeconómico, la cobertura de seguro médico y el nivel educativo, que suelen ser menos favorables en áreas rurales ^26^. Estos elementos configuran un panorama complejo en el que múltiples barreras, tanto individuales como contextuales, interactúan para afectar la salud bucal y el bienestar general de los adultos mayores. 

### Limitaciones

Este estudio presenta varias limitaciones que deben tenerse en cuenta al interpretar los hallazgos. Su diseño transversal impide establecer relaciones causales entre las variables, lo cual limita la determinación de la dirección de los efectos observados. Además, el tamaño muestral reducido, restringido a adultos mayores de una población específica, dificulta la extrapolación de los resultados a otras comunidades rurales del Perú o Sudamérica, que pueden tener contextos socioeconómicos, culturales y geográficos diferentes. La autodeclaración de datos clínicos y sociodemográficos por parte de los participantes puede haber introducido sesgos de memoria o deseabilidad social, y la falta de mediciones objetivas, como evaluaciones clínicas profesionales, reduce la precisión de los diagnósticos, especialmente en afecciones sistémicas y bucales. Asimismo, el contexto rural estudiado, marcado por el aislamiento geográfico y la limitada disponibilidad de servicios de salud, podría no reflejar las condiciones de otras áreas rurales o urbanas. Por ello, los hallazgos deben interpretarse con cautela y no generalizarse sin considerar estas particularidades.

Finalmente, este trabajo destaca importantes fortalezas, como el uso de instrumentos validados (MDAS y OHIP-5) y modelos estadísticos robustos que permitieron identificar asociaciones significativas. Los resultados resaltan la necesidad de diseñar intervenciones específicas para abordar la ansiedad dental y las barreras de acceso a la atención odontológica, a fin de mejorar la salud bucal y la calidad de vida de los adultos mayores en áreas rurales. Este estudio constituye una base sólida para futuras investigaciones y para el desarrollo de políticas públicas dirigidas a reducir inequidades en salud bucal.

## CONCLUSIONES

La ansiedad dental impacta significativamente la calidad de vida relacionada con la salud bucal en adultos mayores rurales, agravada por barreras de acceso y experiencias negativas con la atención odontológica. Este estudio subraya la necesidad de implementar intervenciones que integren estrategias educativas, de accesibilidad y de capacitación profesional, y promuevan así un envejecimiento saludable en esta población vulnerable.
